# Correction: Development and validation of the MMCD score to predict kidney replacement therapy in COVID-19 patients

**DOI:** 10.1186/s12916-023-02912-9

**Published:** 2023-06-06

**Authors:** Flávio de Azevedo Figueiredo, Lucas Emanuel Ferreira Ramos, Rafael Tavares Silva, Daniela Ponce, Rafael Lima Rodrigues de Carvalho, Alexandre Vargas Schwarzbold, Amanda de Oliveira Maurílio, Ana Luiza Bahia Alves Scotton, Andresa Fontoura Garbini, Bárbara Lopes Farace, Bárbara Machado Garcia, Carla Thais Cândida Alves da Silva, Christiane Corrêa Rodrigues Cimini, Cíntia Alcantara de Carvalho, Cristiane dos Santos Dias, Daniel Vitório Silveira, Euler Roberto Fernandes Manenti, Evelin Paola de Almeida Cenci, Fernando Anschau, Fernando Graça Aranha, Filipe Carrilho de Aguiar, Frederico Bartolazzi, Giovanna Grunewald Vietta, Guilherme Fagundes Nascimento, Helena Carolina Noal, Helena Duani, Heloisa Reniers Vianna, Henrique Cerqueira Guimarães, Joice Coutinho de Alvarenga, José Miguel Chatkin, Júlia Drumond Parreiras de Morais, Juliana Machado-Rugolo, Karen Brasil Ruschel, Karina Paula Medeiros Prado Martins, Luanna Silva Monteiro Menezes, Luciana Siuves Ferreira Couto, Luís César de Castro, Luiz Antônio Nasi, Máderson Alvares de Souza Cabral, Maiara Anschau Floriani, Maíra Dias Souza, Maira Viana Rego Souza-Silva, Marcelo Carneiro, Mariana Frizzo de Godoy, Maria Aparecida Camargos Bicalho, Maria Clara Pontello Barbosa Lima, Márlon Juliano Romero Aliberti, Matheus Carvalho Alves Nogueira, Matheus Fernandes Lopes Martins, Milton Henriques Guimarães-Júnior, Natália da Cunha Severino Sampaio, Neimy Ramos de Oliveira, Patricia Klarmann Ziegelmann, Pedro Guido Soares Andrade, Pedro Ledic Assaf, Petrônio José de Lima Martelli, Polianna Delfino-Pereira, Raphael Castro Martins, Rochele Mosmann Menezes, Saionara Cristina Francisco, Silvia Ferreira Araújo, Talita Fischer Oliveira, Thainara Conceição de Oliveira, Thaís Lorenna Souza Sales, Thiago Junqueira Avelino-Silva, Yuri Carlotto Ramires, Magda Carvalho Pires, Milena Soriano Marcolino

**Affiliations:** 1 Department of Internal Medicine, Medical School, Universidade Federal de Minas Gerais, Av. Professor Alfredo Balena, Belo Horizonte, 190 Brazil; 2 Department of Medicine, Universidade Federal de Lavras, R. Tomas Antonio Gonzaga, 277, Lavras, Brazil; 3 Department of Statistics, Universidade Federal de Minas Gerais, Av. Presidente Antônio Carlos, Belo Horizonte, 6627 Brazil; 4 Botucatu Medical School, Universidade Estadual Paulista “Júlio de Mesquita Filho”, Av. Prof. Mário Rubens Guimarães Montenegro, S/N, , Botucatu, Brazil; 5 Institute for Health Technology Assessment (IATS/ CNPq), R. Ramiro Barcelos, , Porto Alegre, 2359 Brazil; 6 Hospital Universitário da Universidade Federal de Santa Maria, Av. Roraima, 1000, Santa Maria, Brazil; 7 Hospital São João de Deus, R. Do Cobre, 800, Divinópolis, Brazil; 8 Hospital Regional Antônio Dias, R. Maj. Gote, 1231, Patos de Minas, Brazil; 9 Hospital Nossa Senhora da Conceição and Hospital Cristo Redentor, Av. Francisco Trein, 326, Porto Alegre, Brazil; 10 Hospital Risoleta Tolentino Neves, R. das Gabirobas, 01, Belo Horizonte, Brazil; 11 Hospital Júlia Kubitschek, R. Dr. Cristiano Rezende, 2745, Belo Horizonte, Brazil; 12 Hospital Santo Antônio, Praça Dr. Márcio Carvalho Lopes Filho, 501, Curvelo, Brazil; 13 Hospital Santa Rosália, R. Do Cruzeiro, 01, Teófilo Otoni, Brazil; 14 Mucuri Medical School, Universidade Federal Dos Vales Do Jequitinhonha E Mucuri, R. Cruzeiro, 01, Teófilo Otoni, Brazil; 15 Hospital João XXIII, Av. Professor Alfredo Balena, 400, Belo Horizonte, Brazil; 16 Department of Pediatrics, Medical School, Universidade Federal de Minas Gerais, Av. Professor Alfredo Balena, 190, Belo Horizonte, Brazil; 17 Hospital UNIMED BH, Av. Do Contorno, 3097, Belo Horizonte, Brazil; 18 Hospital Mãe de Deus, R. José de Alencar, 286, Porto Alegre, Brazil; 19 Hospital Universitário Canoas, Av. Farroupilha, 8001, Canoas, Brazil; 20 Hospital SOS Cárdio, Rodovia, SC-401, 121, Florianópolis, Brazil; 21 Hospital das Clínicas da Universidade Federal de Pernambuco, Av. Prof. Moraes Rego, 1235, Recife, Brazil; 22 Medical School and University Hospital, Universidade Federal de Minas Gerais, Avenida Professor Alfredo Balena, Belo Horizonte, 190 Brazil; 23 Hospital Universitário Ciências Médicas, R. Dos Aimorés, 2896, Belo Horizonte, Brazil; 24 Hospital São Lucas da PUCRS, Av. Ipiranga, 6690, Porto Alegre, Brazil; 25 Hospital Luxemburgo, R. Gentios, 1350, Belo Horizonte, Brazil; 26 Hospital Metropolitano Odilon Behrens, R. Formiga, 50, Belo Horizonte, Brazil; 27 Medical School, Universidade Federal de Ouro Preto, R. Diogo de Vasconcelos, 122, Ouro Preto, Brazil; 28 Hospital Bruno Born, Av. Benjamin Constant, 881, Lajeado, Brazil; 29 Hospital Moinhos de Vento, R. Ramiro Barcelos, 910, Porto Alegre, Brazil; 30 Hospital Santa Cruz, R. Fernando Abott, 174, Santa Cruz Do Sul, Brazil; 31 Laboratorio de Investigacao Medica Em Envelhecimento (LIM-66), Serviço de Geriatria, Hospital das Clínicas HCFMUSP, Faculdade de Medicina, Universidade de Sao Paulo, Sao Paulo, Brazil; 32 Research Institute, Hospital Sirio-Libanes, Sao Paulo, Brazil; 33 Hospitais da Rede Mater Dei, Av. Do Contorno, 9000, Belo Horizonte, Brazil; 34 Hospital Márcio Cunha, Av. Kiyoshi Tsunawaki, 48, Ipatinga, Brazil; 35 Hospital Eduardo de Menezes, R. Dr. Cristiano Rezende, 2213, Belo Horizonte, Brazil; 36 Hospital Tacchini, R. Dr. José Mário Mônaco, 358, Bento Gonçalves, Brazil; 37 Hospital Semper, Alameda Ezequiel Dias, 389, Belo Horizonte, Brazil; 38 Hospital Metropolitano Doutor Célio de Castro, R. Dona Luiza, 311, Belo Horizonte, Brazil; 39 Universidade Federal de São João del-Rei, R. Sebastião Gonçalves Coelho, 400, Divinópolis, Brazil; 40 Faculdade Israelita de Ciencias da Saúde Albert Einstein, Hospital Israelita Albert Einstein, Sao Paulo, Brazil; 41 Telehealth Center, University Hospital, Universidade Federal de Minas Gerais, Av. Professor Alfredo Balena, 110, Belo Horizonte, Brazil

**Correction: BMC Med 20, 324 (2022)**

**https://doi.org/10.1186/s12916-022-02503-0**

The original article [1] contained incorrect values in Table 1 which have since been amended.
